# Plasma sclerostin levels are associated with nutritional status and insulin resistance but not hormonal disturbances in women with polycystic ovary syndrome

**DOI:** 10.1007/s00404-020-05656-6

**Published:** 2020-06-26

**Authors:** Katarzyna Wyskida, Grzegorz Franik, Aleksander Jerzy Owczarek, Piotr Choręza, Piotr Kocełak, Paweł Madej, Jerzy Chudek, Magdalena Olszanecka-Glinianowicz

**Affiliations:** 1 Health Promotion and Obesity Management Unit, Department of Pathophysiology, Medical Faculty in Katowice, The Medical University of Silesia, Medyków 18, 40-752 Katowice, Poland; 2 Department of Gynecological Endocrinology, Medical Faculty in Katowice, The Medical University of Silesia, Medyków 14, Katowice, 40-752 Poland; 3 Department of Statistics, Department of Instrumental Analysis, School of Pharmacy with the Division of Laboratory Medicine, Medical University of Silesia, Ostrogórska 30, 41-209 Sosnowiec, Poland; 4 Pathophysiology Unit, Department of Pathophysiology, Medical Faculty in Katowice, The Medical University of Silesia, Medyków 18, Katowice, 40-752 Poland; 5 Department of Internal Medicine and Oncological Chemotherapy, Medical Faculty in Katowice, The Medical University of Silesia, Reymonta 8, Katowice, 40-027 Poland

**Keywords:** Sclerostin, Nutritional status, Insulin resistance, Polycystic ovary syndrome

## Abstract

**Objective:**

The aim of this study was to evaluate the circulating sclerostin levels with nutritional status, insulin resistance and hormonal disturbances in women with polycystic ovary syndrome (PCOS).

**Patients and methods:**

The cross-sectional study involved 98 PCOS inpatients (20 normal weight, 17 overweight and 61 obese) with stable body mass. Body composition was assessed by bioimpedance method in addition to anthropometric measurements (body mass and height). Serum/plasma concentrations of glucose, insulin (with the calculation of homeostatic model assessment insulin resistance—HOMA-IR), estradiol, total testosterone, sex hormone-binding globulin (SHBG) and sclerostin were measured. Free androgen index (FAI) and estradiol/testosterone index were calculated.

**Results:**

Plasma sclerostin levels were significantly higher in obese [0.61 (interquartile range 0.53–0.77) ng/mL] than in overweight [0.53 (0.49–0.57) ng/mL] and normal weight [0.49 (0.42–0.54) ng/mL] groups. Plasma sclerostin levels were significantly higher in the subgroup with insulin resistance [0.65 (interquartile range 0.53–0.77) vs. 0.52 (0.46–0.58) ng/mL; *p* < 0.001], while similar concentrations were observed in subgroups with FAI below and above median. Plasma sclerostin levels variability were explained by BMI (*r* = 0.40), the percentage of body fat (*r* = 0.40) and HOMA-IR values (*r* = 0.34) in multivariable models.

**Conclusions:**

Circulating sclerostin levels in women with PCOS are related to nutritional status and insulin resistance, but not to sex hormone disturbances.

## Introduction

Sclerostin is a protein member of DAN family that includes Wise, CCN [cysteine-rich 61 (Cyr61, CCN1), connective tissue growth factor (CTGF, CCN2) and nephroblastoma overexpressed (Nov, CCN3)], Dan (differential screening-selected gene aberrant in neuroblastoma), VWF (von Willebrand factor), Norrin, Mucin and Slits [1]. This protein is secreted by osteocytes and participates in the regulation of bone turnover, preventing excessive bone formation [2]. The role of sclerostin is inhibition of the Wnt signaling pathway engaged in the differentiation of osteoblasts [3]. Several factors modulate transduction of sclerostin signaling, e.g. calcitriol [4], parathyroid hormone (PTH) [5], glucocorticoids [6] and tumor necrosis factor-α (TNF-α) in osteoblasts [7]. Circulating sclerostin levels increase with age in both genders, but the increase is almost higher twice in males compared to females, which may be a reason for impaired bone formation during aging [8].

Results of the experimental study showed that Sost^−/−^ mice, in addition to a dramatic increase in bone volume, have reduced accumulation of adipose tissue, but increased insulin sensitivity [9]. Moreover, Sost^−/−^ mice and those administered a sclerostin-neutralizing antibody are resistant to obesogenic diet-induced disturbances in metabolism as the effect on Wnt signaling [10]. Amrein et al. [11] revealed that in healthy subjects sclerostin levels correlate positively with age, BMI, WHR and bone mineral content, and negatively with serum osteocalcin and calcium concentrations. Besides, higher sclerostin levels were found in subjects with prediabetes, as well as with type 1 and 2 diabetes [12–14], and a positive correlation between sclerostin levels and insulin resistance was showed [12]. On the contrary, two studies have shown an increase in sclerostin levels after diet-induced weight loss [15, 16].

A list of factors affecting sclerostin production is longer and includes sex hormones. It was found that sclerostin levels are inversely related to serum estradiol (E_2_) in early postmenopausal women [17], while in men was proportional to testosterone levels [18].

Polycystic ovary syndrome (PCOS) is an endocrine–metabolic disturbance, characterized by hyperandrogenism and insulin resistance and potentially may be associated with an increased prevalence of osteoporosis [19]. It is currently unknown whether the production of sclerostin is disturbed in women with PCOS.

Already described hormonal abnormalities in women with PCOS predisposing to lower bone mineral density (BMD) and the increased risk of the development of osteoporosis include amenorrhea, hypovitaminosis D, low growth hormone level and hypercortisolemia [20]. A recent meta-analysis showed that women with PCOS and BMI values below 27 kg/m^2^ had lower BMD values of total femur and spine than controls, whereas females with BMI values over 27 kg/m^2^ did not present any differences in BMD when compared to controls [21]. Also, long-term studies showed no difference in the risk of fracture between women with PCOS and controls [22] or even a substantial reduction in the risk in PCOS [23].

So far, a 24-h profile of sclerostin secretion has been evaluated in a single study, in men only. This study showed no diurnal changes in sclerostin levels [24]. However, to exclude the potential effect of a glucose load, sclerostin levels are measured in fasting state [25].

The aim of this study was to evaluate the circulating sclerostin levels to nutritional status, insulin resistance and hormonal disturbances in women with PCOS.

## Materials and methods

This cross-sectional, retrospective study involved non-selected inpatient women with PCOS diagnosed according to Rotterdam ESHRE/ASRM criteria from 2003 [26] with stable body mass during the last 3-month period. Any pharmacological therapy, smoking and alcohol abuse were among the exclusion criteria. Ninety-eight women (20 normal weight, 17 overweight and 61 obese) met the criteria type A PCOS [secondary amenorrhea, clinical and biochemical features of hyperandrogenism and polycystic ovary in ultrasound (GE Healthcare Voluson 730 Expert)] in the period from 2015 to 2018 and were tested within 3 and 5 days of the menstrual cycle during short diagnostic hospitalization in the Department of Gynecological Endocrinology. Clinical characteristics’ of study subgroups is presented in Table 1. The study protocol was approved by the Institutional Bioethical Committee. The examination was conducted after obtaining informed consent from each participant.

**Table 1:** Clinical characteristics of study groups

	Normal weight *N* = 20	Overweight *N* = 17	Obese *N* = 61
Hirsutism [*N*/%] score ≥ 8-point Ferriman and Gallwey scale	4 (20.0)	9 (52.9)	48 (78.7)
Acne [*N*/%] on the basis global scale of acne severity	9 (45.0)	8 (47.1)	26 (42.6)
Androgenetic alopecia [*N*/%]	0	0	0
Dysmenorrhea [*N*/%]	16 (80.0)	11 (64.7)	27 (44.3)
Secondary amenorrhea [*N*/%]	4 (20.0)	6 (35.3)	34 (55.7)
Polycystic ovary morphology in ultrasound [*N*/%]	20 (100)	17 (100)	61 (100)

Anthropometric measurements (body mass, height and waist circumference) were taken, and body composition was assessed by bioimpedance method using Bodystat 1500 (Douglas, Isle of Man). BMI was calculated according to the standard formula. Venous blood samples (15 ml) were withdrawn in the morning between 8.00–9.00 a.m., after an overnight prolonged fast. Serum and plasma samples (collected according to the recommendation of the manufacturer of the kits) were stored frozen in − 70 °C.

### Biochemical measurements

Plasma sclerostin levels were measured by ELISA (TECOmedical AG, Sissach, Switzerland) with the mean intra- and interassay coefficients < 4.0% and the < 4.8%, respectively. Serum concentrations of insulin, estradiol (E_2_), total testosterone (T) and sex hormone-binding globulin (SHBG) were determined by electro-chemiluminescence immunoassay (ECLIA) using Cobas E411 analyzer (Roche Diagnostics GmbH, Mannheim, Germany) with intra- and interassay coefficients of variations of 2.0% and 2.8% for insulin, 4.6% and 9.9% for E_2_, 4.7% and 8.4% for T and 2.7% and 5.6% for SHBG, respectively. Serum glucose was estimated by colorimetric methods using the commercially available test kits (Roche, Switzerland). All the methods were performed following the relevant guidelines.

### Data analysis

In accordance with WHO criteria, normal weight was defined as body mass index (BMI) from 18.5 to 24.9 kg/m^2^, overweight from 25.0 to 29.9 kg/m^2^ and obesity as ≥ 30.0 kg/m^2^.

HOMA-IR index was calculated with the standard formula: HOMA-IR = fasting concentration of insulin (μIU/mL) x fasting concentration of glucose (mg/dL) / 405. The free androgen index (FAI) was calculated according to the standard formula [(T/SHBG) × 100]. Insulin resistance was defined as HOMA-IR values ≥ 2.0 [27].

### Statistical analysis

Statistical analysis was performed with Statistica 12.0 software (TIBCO Software Inc., Palo Alto, California, USA). Nominal and ordinal data were expressed as percentages, while interval data were expressed as mean value ± standard deviation in case of a normal distribution or as median with lower and upper quartile in case of data with skewed or non-normal distribution. Distribution of variables was evaluated by the Shapiro–Wilk test and quantile–quantile (Q–Q) plot. For comparison of data between normal, overweight and obese groups, the one-way analysis of variance (ANOVA) was used with Tukey post hoc test. For comparison of data between the group with and without insulin resistance (HOMA-IR ≥ 2 vs. < 2), the Student* t* test for independent data (in case of normal data distribution or after logarithmic normalization if appropriate—in case of skewed distribution) or the nonparametric *U* Mann–Whitney test (in non-normal data distribution) was used. The Pearson correlation coefficient was used as a measure of association between analyzed variables. Multivariable stepwise backward regression analysis was performed for sclerostin serum levels as an independent variable with potentially explanatory variables: body mass index (BMI) (model I), % of fat mass (model II), HOMA-IR (model III) and serum levels of insulin (except model III), E_2_, FAI and age. Such three models were used due to high level of multicollinearity between log_10_(BMI) and percentage of fat mass (*r* = 0.93) and log_10_(HOMA-IR) (*r* = 0.57) as well as between percentage of fat mass and log_10_(HOMA-IR) (*r* = 0.51). The Cook–Weisberg test was used to test heteroskedasticity, and the Ramsey RESET test was used to test the linearity of regression. The variance inflation factor VIF was calculated to check multicollinearity. The goodness of fit of the obtained regression models was assessed with the adjusted determination coefficient *R*^2^. All tests were two-tailed. The results were considered statistically significant with a *p* value of less than 0.05.

## Results

The characteristics of the study groups are presented in Table 1. Plasma sclerostin levels were significantly higher in obese than in normal weight and overweight groups. Besides, the obese group was also characterized by significantly higher insulin and E_2_ serum levels, HOMA-IR values and index of E_2_/T values. FAI values were significantly higher in both obese and overweight groups than the normal weight group, but did not differ between obese and overweight groups (Table 2).

**Table 2:** Characteristics of study groups divided according to BMI values (values are presented as means with standard deviation or median with interquartile range in parentheses)

	Normal weight	Overweight	Obese	*p*	*p* I vs II	*p* I vs III	*p* II vs III
*N* [%]	20 (20.4)	17 (17.3)	61 (62.3)	–	–	–	–
Age [years]	24 ± 4	27 ± 5	28 ± 6	< 0.05	0.16	< 0.05	0.98
Body mass [kg]	53.7 ± 5.5	75.8 ± 5.3	107.1 ± 16.6	< 0.001	< 0.001	< 0.001	< 0.001
BMI [kg/m^2^]	19.3 (18.3–20.7)	27.7 (27.1–29.1)	38.3 (34.8–41.7)	< 0.001	< 0.001	< 0.001	< 0.001
Fat percentage [%]	28.6 ± 7.1	37.2 ± 4.4	48.9 ± 5.1	< 0.001	< 0.001	< 0.001	< 0.001
Fat mass [kg]	15.8 (11.4–18.0)	29.4 (24.9–30.7)	50.5 (45.5–58.7)	< 0.001	< 0.001	< 0.001	< 0.001
Waist circumference [cm]	68.6 ± 6.1	85.4 ± 8.7	108.8 ± 12.5	< 0.001	< 0.001	< 0.001	< 0.001
Glucose [mg/dL]	91.7 ± 11.4	89..1 ± 12.6	90.5 ± 10.9	0.74	–	–	–
Insulin [uIU/mL]	8.6 (4.7–12.4)	11.4 (8.2–15.9)	21.3 (15.8–29.0)	< 0.001	0.19	< 0.001	< 0.01
HOMA-IR	1.7 (1.0–2.9)	2.5 (2.2–3.0)	4.8 (3.5–6.5)	< 0.001	0.30	< 0.001	< 0.01
Estradiol–E_2_ [pg/mL]	167 (113–219)	161 (83–309)	81 (61–120)	< 0.01	0.84	0.12	< 0.05
Testosterone—T [ng/mL]	0.37 ± 0.20	0.36 ± 0.16	0.40 ± 0.20	0.69	–	–	–
SHBG [nmol/L]	81.6 (58.7–107.6)	32.8 (27.3–62.7)	26.5 (20.1–39.0)	< 0.001	< 0.001	< 0.001	0.38
Free androgen index (FAI)	0.5 (0.3–0.7)	1.1 (0.5–2.0)	1.4 (0.8–2.3)	< 0.001	< 0.05	< 0.01	0.66
E_2_/T index	475 (314–695)	481 (340–850)	249 (154–469)	< 0.01	0.98	< 0.05	< 0.05
Sclerostin [ng/mL]	0.49 (0.42–0.54)	0.53 (0.49–0.57)	0.61 (0.53–0.77)	< 0.001	0.48	< 0.01	< 0.05

I vs. II normal weight vs overweight group

I vs. II normal weight vs obese group

II vs. III overweight vs obese group

An analysis performed in groups with and without insulin resistance (HOMA-IR ≥ 2 vs. < 2) showed that plasma sclerostin levels were significantly higher in the group with insulin resistance. This group was also characterized by significantly higher testosterone levels and FAI values, lower SHBG levels and E_2_/T index values (Table 3).

**Table 3:** Characteristics of study groups divided according to HOMA-IR value defining insulin resistance

	HOMA-IR			FAI		
	< 2.0	≥ 2.0	*p*	< 1.04	≥ 1.04	*p*
*N* (%)	19 (19.4%)	79 (80.6%)	–	49 (50%)	49 (50%)	–
Age [years]	27 ± 7	27 ± 6	0.90	28 ± 6	26 ± 6	0.15
Body mass [kg]	67.1 ± 17.0	96.4 ± 24.5	< 0.001	80.1 ± 26.3	101.4 ± 20.8	< 0.001
BMI [kg/m^2^]	23.2 (19.3–27.9)	35.5 (29.4–40.8)	< 0.001	28.6 (20.6–34.8)	36.8 (30.6–40.8)	< 0.001
Fat mass [%]	33.8 ± 10.1	44.9 ± 8.7	< 0.001	40.0 ± 11.6	45.5 ± 7.3	< 0.01
Fat percentage [kg]	21.7 (15.7–29.9)	47.0 (32.9–55.4)	< 0.001	32.8 (17.3–47.0)	48.1 (35.4–55.6)	< 0.001
Waist circumference [cm]	76.9 ± 12.8	101.2 ± 18.3	< 0.001	87.3 ± 19.1	105.7 ± 16.0	< 0.001
Glucose [ng/mL]	87.6 ± 8.0	91.2 ± 11.8	0.21	90.6 ± 10.3	90.4 ± 12.1	0.92
Insulin [uIU/mL]	6.84 (4.41–7.52)	19.02 (13.93–26.70)	< 0.001	12.5 (7.5–17.7)	20.8 (15.9–32.2)	< 0.001
HOMA-IR	–	–	**–**	2.56 (1.64 – 3.76)	4.61 (3.49 – 7.04)	< 0.001
Estradiol–E_2_ [pg/mL]	120.0 (59.6–195.7)	95.4 (64.4 – 208.5)	0.90	120 (69–208)	84 (64–145)	0.40
Testosterone–T [ng/mL]	0.29 ± 0.20	0.41 ± 0.19	< 0.05	0.29 ± 0.16	0.48 ± 0.18	< 0.001
SHBG [nmol/L]	75.5 (41.6–98.7)	29.6 (20.5–53.8)	< 0.001	62.7 (39.5–90.3)	22.5 (18.8–27.8)	< 0.001
Free androgen index	0.40 (0.20–0.61)	1.31 (0.68–2.03)	< 0.001	0.43 (0.29–0.71)	1.89 (1.41–2.52)	**–**
E_2_/T index	489 (335–751)	287 (178–607)	< 0.05	469 (292–751)	227 (136–448)	< 0.001
Sclerostin [ng/mL]	0.49 (0.41–0.58)	0.58 (0.51–0.73)	< 0.001	0.5 (0.47–0.67)	0.58 (0.51–0.73)	0.11

The free androgen index (FAI) medians (values are presented as means with standard deviation or median with interquartile range in parentheses)

The study group was also divided according to FAI value. The levels of sclerostin were similar in both groups, regardless of substantial differences in the levels of testosterone and SHBG, as well as values of E_2_/T index and HOMA-IR (Table 3).

### Multivariable analyses

**Fig. 1:**
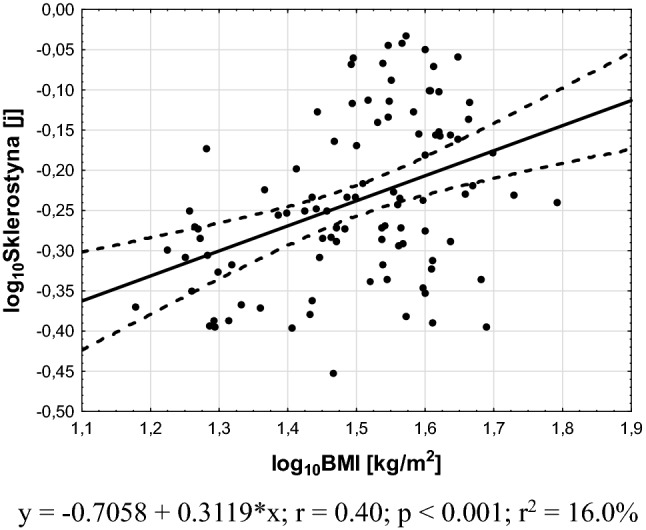
Association between plasma sclerostin levels and BMI values

**Fig. 2:**
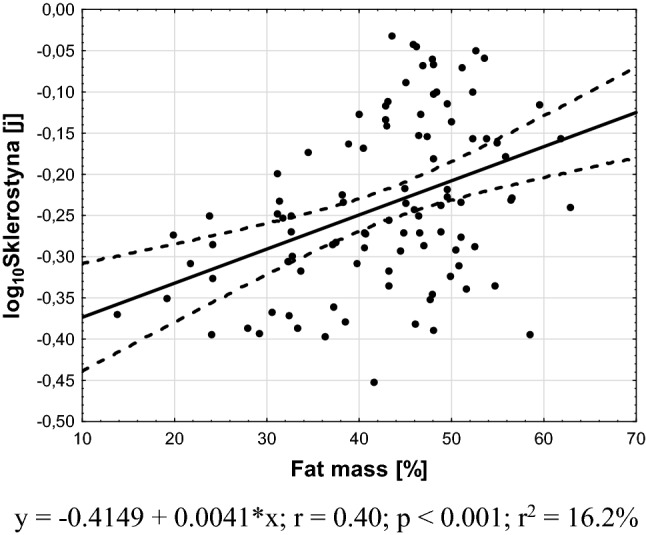
Association between plasma sclerostin levels and fat percentage

**Fig. 3:**
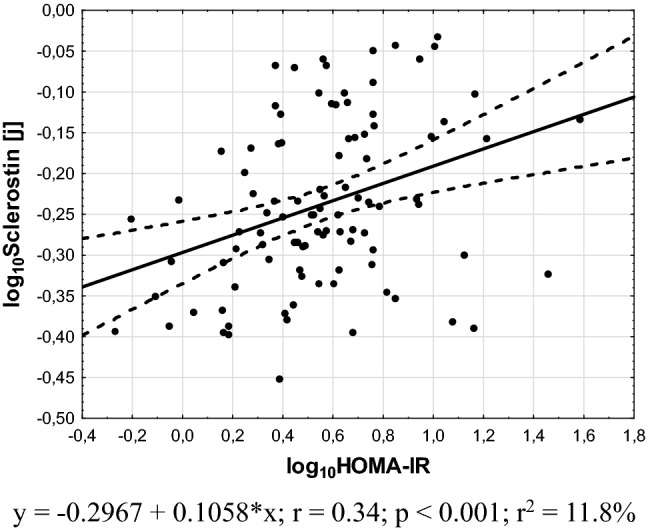
Association between plasma sclerostin levels and HOMA-IR values

For each described in the statistical analysis section regression models, there was only a significant positive correlation between plasma sclerostin levels and BMI (model 1), the percentage of body fat (model 2) and HOMA-IR values (model 3) (Figs. 1, 2, 3). All other variables taken into account in initial models proved to be non-significant.

## Discussion

To the best our knowledge, this is a first study that assessed the effect of nutritional status, insulin resistance and hormonal disturbances on circulating sclerostin levels in women with PCOS. In addition to significantly higher plasma sclerostin levels in obese than in normal weight and overweight women, we showed that plasma sclerostin variability is explained by nutritional status assessed based on BMI and body fat content, and HOMA-IR, but not FAI values.

As could be expected [11], plasma sclerostin levels positively correlated with BMI, probably reflecting higher bone mass in obese subjects. This hypothesis is supported by results of a large study with 3500 subjects (men, pre- and postmenopausal women) that shows an association between bone mineral density and both lean and fat mass in men and premenopausal women only [28].

An important issue revealed by our study is independent of BMI positive association between sclerostin levels and HOMA-IR values. It is consistent with results obtained in non-PCOS subjects with prediabetes condition and subjects with type 1 and 2 diabetes [12–14]. Notwithstanding a study performed among children showed a negative correlation between sclerostin levels and insulin resistance [29]. These authors suggested that sclerostin plays an important role in the regulation of glucose metabolism regardless of other fat and bone-derived factors and decreased insulin resistance [29]. However, the potential pathomechanism of sclerostin action on glucose metabolism is unknown. The altered Wnt pathway was observed in subcutaneous and visceral white adipose tissue of obese subjects, and its inhibition was associated with insulin resistance [30]. Besides, the experimental study revealed that Sost^−/−^ mice and those administered sclerostin-neutralizing antibodies are resistant to obesogenic diet-induced disturbances in metabolism as the effect on Wnt signaling [10]. But the cohort study performed among 1778 subjects without a history of type 2 diabetes did not confirm the association between sclerostin levels and risk of type 2 diabetes [31]. This suggests that observed association between sclerostin levels and insulin resistance can indirectly reflect the deteriorative effect of higher BMI on insulin sensitivity. Further studies are necessary to explain this hypothesis.

Contrary to a previously published study that showed a negative correlation between sclerostin and estradiol levels in early postmenopausal women [17], we did not observe an association between sclerostin and estradiol levels in women with PCOS. We also failed to find an association between sclerostin and testosterone levels as well as FAI values. Probably the androgen levels in the majority of PCOS are too low to stimulate sclerostin production, as observed in men [18].

Our study suggests that sclerostin levels are not a prognostic marker for hormonal changes specific to PCOS. It seems that they may be a marker for the risk prediabetes and type 2 diabetes development. However, this requires a few-year observational study that would allow determining whether women with a diagnosis of PCOS with normal glucose levels at the time of diagnosis, higher sclerostin levels will be a predictor of prediabetes development.

The main limitation of the present study is the small sample size. The second limitation is the assessment of body composition on the basis bioimpedance method and not DXA method; therefore, the assessment of visceral fat depot was possible only indirectly based on the waist circumference measurement and the lack of assessment of bone density. However, it should be noted that our study is the first study assessing the association between sclerostin levels and nutritional status, insulin resistance and hormone levels in young women with PCOS.

## Conclusions

Circulating sclerostin levels in women with PCOS is related to nutritional status and insulin resistance, but not to sex hormone disturbances.

## References

[1] Ellies DL, Viviano B, McCarthy J (1999). Bone density ligand, Sclerostin, directly interacts with LRP5 but not LRP5G171V to modulate Wnt activity. J Bone Miner Res.

[2] Sevetson B, Taylor S, Pan Y (2004). Cbfa1/RUNX2 directs specific expression of the sclerosteosis gene (SOST). J Biol Chem.

[3] Gaudio A, Privitera F, Battaglia K, Torrisi V, Sidoti MH, Pulvirenti CE, Tringali G, Fiore CE (2012). Sclerostin levels associated with inhibition of the Wnt/b-catenin signalling and reduced bone turnover in type 2 diabetes mellitus. J Clin Endocrinol Metab.

[4] Wijenayaka AR, Yang D, Prideaux M (2015). 1α,25-dihydroxy vitamin D stimulates human SOST gene expression and sclerostin secretion. Mol Cell Endocrinol.

[5] Keller H, Kneissel M (2005). SOST is a target gene for PTH in bone. Bone.

[6] Sato AY, Cregor M, Delgado-Calle J (2016). Protection from glucocorticoid-induced osteoporosis by anti-catabolic signaling in the absence of sost/sclerostin. J Bone Miner Res.

[7] Vincent C, Findlay DM, Welldon KJ (2009). Pro-inflammatory cytokines TNF-related weak inducer of apoptosis (TWEAK) and TNF-alpha induce the mitogen-activated protein kinase (MAPK)-dependent expression of sclerostin in human osteoblasts. J Bone Miner Res.

[8] Modder UI, Hoey KA, Amin S (2011). Relation of age, gender, and bone mass to circulating sclerostin levels in women and men. J Bone Miner Res.

[9] Lin C, Jiang X, Dai Z (2009). Sclerostin mediates bone response to mechanical unloading through antagonizing Wnt/beta-catenin signalling. J Bone Miner Res.

[10] Kim SP, Frey JL, Li Z (2017). Sclerostin influences body composition by regulating catabolic and anabolic metabolism in adipocytes. Proc Natl Acad Sci USA.

[11] Amrein K, Amrein S, Drexler C (2012). Sclerostin and its association with physical activity, age, gender, body composition, and bone mineral content in healthy adults. J Clin Endocrinol Metab.

[12] Daniele G, Winnier D, Mari A (2015). Sclerostin and insulin resistance in prediabetes: evidence of cross-talk between bone and glucose metabolism. Diabetes Care.

[13] Neumann T, Hofbauer LC, Rauner M (2014). Clinical and endocrine correlates of circulating sclerostin levels in patients with type 1 diabetes mellitus. Clin Endocrinol (Oxf).

[14] García-Martín A, Rozas-Moreno P, Reyes-García R (2012). Circulating levels of sclerostin are increased in patients with type 2 diabetes mellitus. J Clin Endocrinol Metab.

[15] Armamento-Villareal R, Sadler C, Napoli N (2012). Weight loss in obese older adults increases serum sclerostin and impairs hip geometry but both are prevented by exercise training. JBMR.

[16] Strollo R, Soare A, Manon Khazrai Y (2017). Increased sclerostin and bone turnover after diet-induced weight loss in type 2 diabetes: a post hoc analysis of the MADIAB trial. Endocrine.

[17] Matsui S, Yasui T, Kasai K (2016). Increase in circulating sclerostin at the early stage of the menopausal transition in Japanese women. Maturitas.

[18] Modder UI, Clowes JA, Hoey K (2011). Regulation of circulating sclerostin levels by sex steroids in women and men. J Bone Miner Res.

[19] Krishnan A, Muthusami S (2017). Hormonal alterations in PCOS and its influence on bone metabolism. J Endocrinol.

[20] Glintborg D, Hermann AP, Andersen M (2013). Bone mineral density and vitamin D in PCOS and hirsutism. Expert Rev Endocrinol Metab.

[21] Mottecy Piovezan J, Orlandin Premaor M, Vasconcellos Comim F (2019). Negative impact of polycystic ovary syndrome on bone health: a systematic review and meta-analysis. Hum Reprod Update.

[22] Schmidt J, Dahlgren E, Brannstrom M, Landin-Wilhelmsen K (2012). Body composition, bone mineral density and fractures in late postmenopausal women with polycystic ovary syndrome - a long-term follow-up study. Clin Endocrinol (Oxf).

[23] Hass Rubin K, Glintborg D, Nybo M, Andersen M, Abrahamsen B (2016). Fracture risk is decreased in women with polycystic ovary syndrome: a register-based and population-based cohort study. J Bone Miner Res.

[24] Swanson C, Shea SA, Wolfe P (2017). 24-Hour profile of serum sclerostin and its association with bone biomarkers in men. Osteoporos Int.

[25] Shimizu M, Onoe Y, Mikumo M (2009). Variations in circulating osteoprotegerin and soluble RANKL during diurnal and menstrual cycles in young women. Hormone Res.

[26] Rotterdam ESHRE/ASRM-Sponsored PCOS Consensus Workshop Group (2004). Revised 2003 consensus on diagnostic criteria and long-term health risks related to polycystic ovary syndrome. Fertil Steril.

[27] Wongwananuruk T, Rattanachaiyanont M, Leerasiri P (2012). The usefulness of Homeostatic Measurement Assessment-Insulin Resistance (HOMA-IR) for detection of glucose intolerance in Thai women of reproductive age with Polycystic Ovary Syndrome. Int J Endocrinol.

[28] Lee SJ, Lee JY, Sung J (2019). Obesity and bone health revisited: a mendelian randomization study for Koreans. J Bone Miner Res.

[29] Wedrychowicz A, Sztefko K, Starzyk JB (2018). Sclerostin and its association with insulin resistance in children and adolescents. Bone.

[30] Ehrlund A, Mejhert N, Lorente-Cebrián S (2013). Characterization of the Wnt inhibitors secreted frizzled-related proteins (SFRPs) in human adipose tissue. J Clin Endocrinol Metab.

[31] Yu OH, Richards B, Berger C (2017). The association between sclerostin and incident type 2 diabetes risk: a cohort study. Clin Endocrinol (Oxf).

